# Correction: GPT-4 can pass the Korean National Licensing Examination for Korean Medicine Doctors

**DOI:** 10.1371/journal.pdig.0001000

**Published:** 2025-09-03

**Authors:** Dongyeop Jang, Taerim Yun, Choong-Yeol Lee, Young-Kyu Kwon, Chang-Eop Kim

The second author’s name is spelled incorrectly. The correct name is: Taerim Yun. The correct citation is: Jang D, Yun T, Lee C-Y, Kwon Y-K, Kim C-E (2023) GPT-4 can pass the Korean National Licensing Examination for Korean Medicine Doctors. PLOS Digit Health 2(12): e0000416. https://doi.org/10.1371/journal.pdig.0000416

The ORCID iDs are missing for the second and third author. Please see the authors’ respective ORCID iDs here:

- Author Taerim Yun’s ORCID iD is: 0009-0004-1663-0485 (https://orcid.org/0009-0004-1663-0485).
- Author Choong-Yeol Lee’s ORCID iD is: 0000-0001-8441-2675 (https://orcid.org/0000-0001-8441-2675).

## References

[1] Jang D, Yun T-R, Lee C-Y, Kwon Y-K, Kim C-E. GPT-4 can pass the Korean National Licensing Examination for Korean Medicine Doctors. PLOS Digit Health. 2023;2(12):e0000416. doi: 10.1371/journal.pdig.0000416 38100393 PMC10723673

