# Supplementary material for: GPT-4 can pass the Korean National Licensing Examination for Korean Medicine Doctors
Source: PLOS Digit Health. 2023 Dec 15;2(12):e0000416. doi: 10.1371/journal.pdig.0000416 (PMC10723673; doi:10.1371/journal.pdig.0000416)
Supplement: S1 Fig — Note that the results shown in this figure are for the performance of the model when different prompting techniques including translating the question into English, providing exam-optimized instructions, and utilizing a self-consistency in the prompt were not used. The x-axis and y-axis represent the language model and its accuracy, respectively. The heights of the bars represent the mean of accuracy for multiple trials. The color represents the model (GPT-4 or GPT-3.5). A circle mark indicates accuracy in each trial. Dashed lines indicate the chance level of accuracy (= 0.2) and the pass mark (= 0.6). (DOCX) [file pdig.0000416.s001.docx]

**
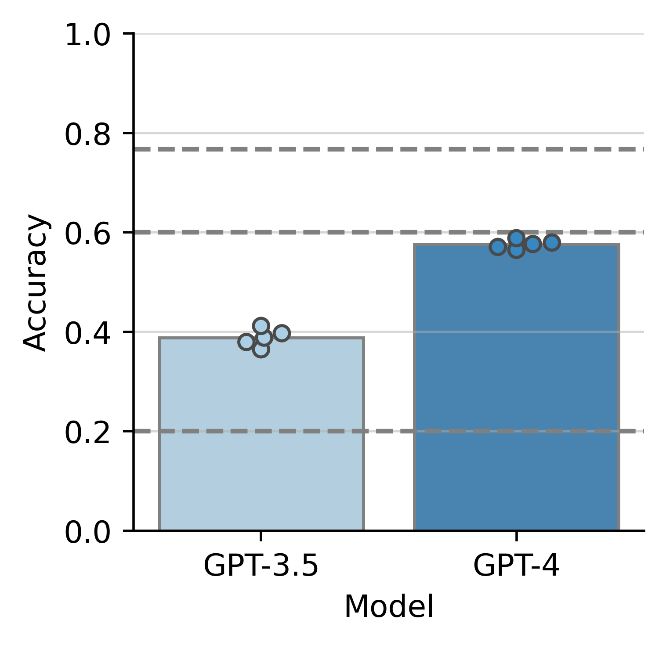
**

Supplementary Figure 1. The overall performance of the GPT-3.5 and GPT-4 with Chinese-term annotation. Note that the results shown in this figure are for the performance of the model when different prompting techniques including translating the question into English, providing exam-optimized instructions, and utilizing a self-consistency in the prompt were not used. The x-axis and y-axis represent the language model and its accuracy, respectively. The heights of the bars represent the mean of accuracy for multiple trials. The color represents the model (GPT-4 or GPT-3.5). A circle mark indicates accuracy in each trial. Dashed lines indicate the chance level of accuracy (= 0.2) and the pass mark (= 0.6).
