# Supplementary material for: GPT-4 can pass the Korean National Licensing Examination for Korean Medicine Doctors
Source: PLOS Digit Health. 2023 Dec 15;2(12):e0000416. doi: 10.1371/journal.pdig.0000416 (PMC10723673; doi:10.1371/journal.pdig.0000416)
Supplement: S2 Fig — The y-axis indicates the subjects the questions are related to, and the x-axis indicates the accuracy on the questions. The colors of the bar indicate whether TKM-specialized knowledge is required to answer for the questions. Other details are the same as in Fig 2. (DOCX) [file pdig.0000416.s002.docx]

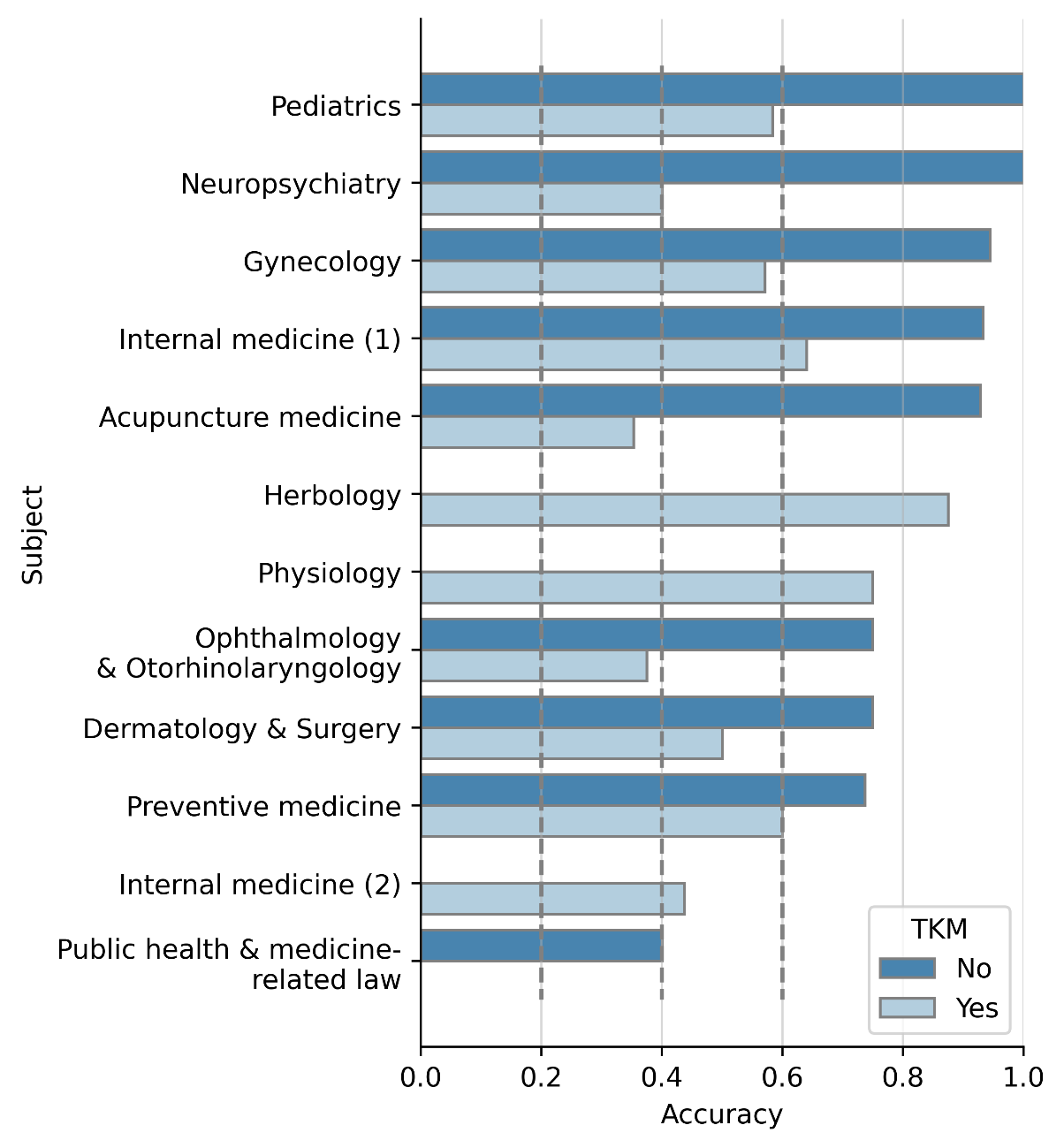


Supplementary Figure 2. The difference in accuracy between subjects. The y-axis indicates the subjects the questions are related to, and the x-axis indicates the accuracy on the questions. The colors of the bar indicate whether TKM-specialized knowledge is required to answer for the questions. Other details are the same as in Figure 2.
