# Supplementary material for: GPT-4 can pass the Korean National Licensing Examination for Korean Medicine Doctors
Source: PLOS Digit Health. 2023 Dec 15;2(12):e0000416. doi: 10.1371/journal.pdig.0000416 (PMC10723673; doi:10.1371/journal.pdig.0000416)
Supplement: S3 Fig — The x-axis indicates what competency types the question is intended to assess, and the y-axis indicates the accuracy on the questions. The colors of the bar indicate whether TKM-specialized knowledge is required to answer for the questions. Other details are the same as in Fig 1. (DOCX) [file pdig.0000416.s003.docx]

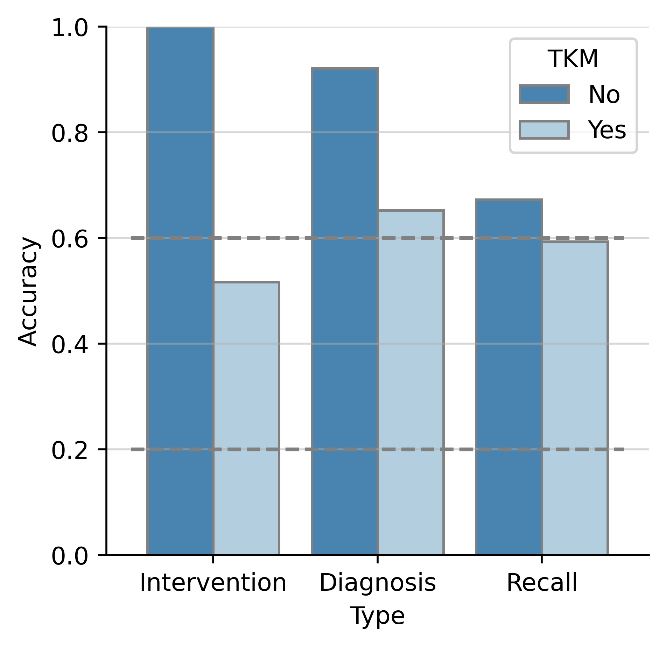


Supplementary Figure 3. The difference in accuracy between questions that require TKM knowledge and questions that do not, compared by competency types. The x-axis indicates what competency types the question is intended to assess, and the y-axis indicates the accuracy on the questions. The colors of the bar indicate whether TKM-specialized knowledge is required to answer for the questions. Other details are the same as in Figure 1.
