# Supplementary material for: GPT-4 can pass the Korean National Licensing Examination for Korean Medicine Doctors
Source: PLOS Digit Health. 2023 Dec 15;2(12):e0000416. doi: 10.1371/journal.pdig.0000416 (PMC10723673; doi:10.1371/journal.pdig.0000416)
Supplement: S5 Fig — Details are the same as in the S2 Fig. (DOCX) [file pdig.0000416.s005.docx]

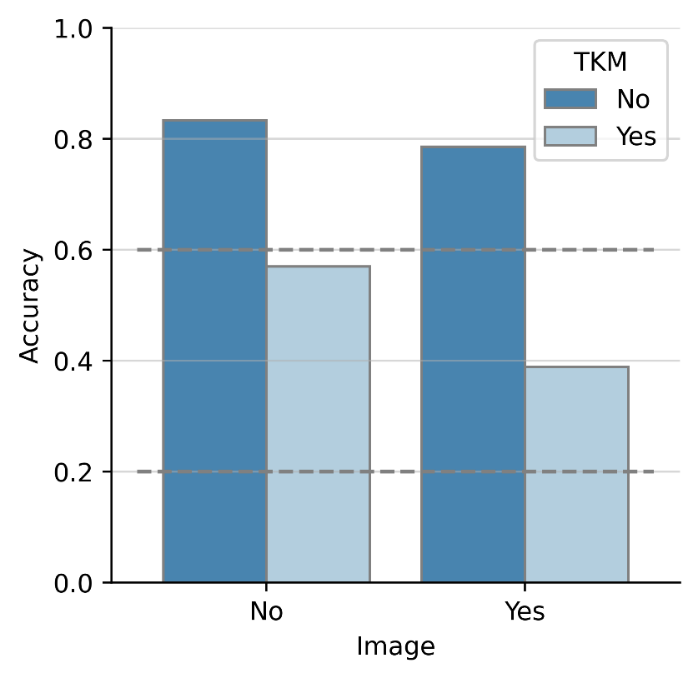


Supplementary Figure 5. The difference in accuracy between questions that require TKM knowledge and questions that do not, compared by whether the question includes images or not. Details are the same as in the Supplementary Figure 2.
