# Supplementary material for: GPT-4 can pass the Korean National Licensing Examination for Korean Medicine Doctors
Source: PLOS Digit Health. 2023 Dec 15;2(12):e0000416. doi: 10.1371/journal.pdig.0000416 (PMC10723673; doi:10.1371/journal.pdig.0000416)
Supplement: S1 Table — (DOCX) [file pdig.0000416.s006.docx]

Supplementary Table 1. Examples for prompt engineering in this study.

| Chinese-term annotation | English -translated instruction | English-translated questions | Exam-optimized | Self-consistency | Prompt text |
| --- | --- | --- | --- | --- | --- |
| No | No | No | No | No | 당신은 중의학 및 한의학 모두에 대한 전문가입니다. 질문에 대한 정답은 무엇인가요? 이 질문의 정답은 하나뿐입니다.:  (question written in Korean) |
| Yes | No | No | No | No | 당신은 중의학 및 한의학 모두에 대한 전문가입니다. 질문에 대한 정답은 무엇인가요? 이 질문의 정답은 하나뿐입니다.:  (question written in Korean with Chinese-annotation) |
| Yes | Yes | No | No | No | You are an expert of both Traditional Chinese medicine and Traditional Korean medicine.  What's the answer to the question?  This question has only one correct answer.:  (question written in Korean with Chinese-annotation) |
| Yes | Yes | Yes | No | No | You are an expert of both Traditional Chinese medicine and Traditional Korean medicine who are very good at multiple languages such as Chinese, Korean, and English. Translate the text below, delimited by three dashes (-), into English, but leave the Chinese characters as they are. Don't say anything but the translated text.  (question written in Korean with Chinese-annotation)  (translate the question and input the translated question with the below prompt text)  You are an expert of both Traditional Chinese medicine and Traditional Korean medicine. What's the answer to the question, delimited by three dashes(-)? This question has only one correct answer.  (question translated in English with Chinese-annotation) |
| Yes | Yes | Yes | Yes | No | You are an expert of both Traditional Chinese medicine and Traditional Korean medicine who are very good at multiple languages such as Chinese, Korean, and English.  Translate the text below, delimited by three dashes (-), into English, but leave the Chinese characters as they are.  Don't say anything but the translated text.  (question written in Korean with Chinese-annotation)  (translate the question and input the translated question with the below prompt text)  You are an expert of both Traditional Chinese medicine and Traditional Korean medicine.  What's the answer to the question, delimited by three dashes(-)?Let's work this out in a step by step way to be sure we have the right answer.  This question has only one correct answer.  Provide the answer in numbers only without any explanation.  In other words, answer should be one of '1','2','3','4','5'.  (question translated in English with Chinese-annotation) |
| Yes | Yes | Yes | Yes | Yes | You are an expert of both Traditional Chinese medicine and Traditional Korean medicine who are very good at multiple languages such as Chinese, Korean, and English.  Translate the text below, delimited by three dashes (-), into English, but leave the Chinese characters as they are.  Don't say anything but the translated text.  (question written in Korean with Chinese-annotation)  (translate the question and input the translated question with the below prompt text)  You are an expert of both Traditional Chinese medicine and Traditional Korean medicine.  What's the answer to the question, delimited by three dashes(-)?  Let's work this out in a step by step way to be sure we have the right answer.  This question has only one correct answer.  Provide the answer in numbers only without any explanation.  In other words, answer should be one of '1','2','3','4','5'.  (question translated in English with Chinese-annotation)  (select the most frequent answer for each question in the multiple test results) |
