# Supplementary material for: GPT-4 can pass the Korean National Licensing Examination for Korean Medicine Doctors
Source: PLOS Digit Health. 2023 Dec 15;2(12):e0000416. doi: 10.1371/journal.pdig.0000416 (PMC10723673; doi:10.1371/journal.pdig.0000416)
Supplement: S2 Table — The original questions were all written in Korean, but we present both original questions written in Korean and translated questions in English for the reader’s convenience. The underlines indicate Chinese-annotated TKM terms. TKM, traditional Korean medicine. (DOCX) [file pdig.0000416.s007.docx]

Supplementary Table 2. An example of an original question and Chinese-annotated question input into GPT-4. Since the questions of the Korean national licensing examination for Korean medicine doctors used in the study are not publicly available, we show virtual questions as an example. The original questions were all written in Korean, but we present both original questions written in Korean and translated questions in English for the reader's convenience. The underlines indicate Chinese-annotated TKM terms. TKM, traditional Korean medicine.

| Original question | 65세 남자 환자가 상복부의 통증을 주증상으로 의원을 방문하였다. 소화기내시경 결과 특별한 병변을 발견하지 못했다. 목소리가 작고 낮으며 식욕부진을 호소한다. 혀는 창백하게 붉고 설태는 얇으며, 맥세하다. 다음 중 가장 적합한 처방은?  ① 사군자탕  ② 사물탕  ③ 도담탕  ④ 억간산  ⑤ 부자이중탕  (translation:  A 65-year-old man visits a clinic with the main complaint of pain in the upper abdomen. Gastroscopy did not reveal any lesions. His voice is low when he speaks and he has no appetite. He has a pale pink tongue with thin fur, and a fine pulse. Which of the following prescriptions is appropriate for the patient?  ① Sijunzi-tang  ② Siwu-tang  ③ Daotan-tang  ④ Yigan-san  ⑤ Fuzilizhong-tang  ) |
| --- | --- |
| Questions with annotation for the Chinese-terms in TKM | 65세 남자 환자가 상복부의 통증을 주증상으로 의원을 방문하였다. 소화기내시경 결과 특별한 병변을 발견하지 못했다. 목소리가 작고 낮으며 식욕부진을 호소한다. 혀는 창백하게 붉고(舌質淡紅) 설태는 얇으며(舌苔薄), 맥세(脈細)하다. 다음 중 가장 적합한 처방은?  ① 사군자탕(四君子湯)  ② 사물탕(四物湯)  ③ 도담탕(導痰湯)  ④ 억간산(抑肝散)  ⑤ 부자이중탕(附子理中湯)  (translation:  A 65-year-old man visits a clinic with the main complaint of pain in the upper abdomen. Gastroscopy did not reveal any lesions. His voice is low when he speaks and he has no appetite. He has a pale pink tongue (舌質淡紅) with thin fur (舌苔薄), and a fine pulse (脈細). Which of the following prescriptions is appropriate for the patient?  ① Sijunzi-tang (四君子湯)  ② Siwu-tang (四物湯)  ③ Daotan-tang (導痰湯)  ④ Yigan-san (抑肝散)  ⑤ Fuzilizhong-tang (附子理中湯)  ) |
