# Supplementary material for: GPT-4 can pass the Korean National Licensing Examination for Korean Medicine Doctors
Source: PLOS Digit Health. 2023 Dec 15;2(12):e0000416. doi: 10.1371/journal.pdig.0000416 (PMC10723673; doi:10.1371/journal.pdig.0000416)
Supplement: Attachment — Submitted filename: Response to Reviewers.docx [file pdig.0000416.s008.docx]

**Dear Editor,**

We are deeply grateful for the constructive feedback from the editors and reviewers. Based on the advice from the editors and reviewers, we were able to significantly improve the quality of the manuscript. Influenced by the advice from reviewer 1 concerning language translation, during our exploration of GPT-4's performance, we found that GPT-4 can actually pass the licensing examination for Korean medicine doctors. We have thus added and emphasized these findings and accordingly, we made some changes in the way we presented the results.

We have opted to relocate the results pertaining to GPT-3.5 to the supplementary section. Instead, we have incorporated a more comprehensive analysis of GPT-4's results, responding to the valuable feedback from the reviewers. Given the swift progression in this domain, we feel that GPT-3.5 may no longer be at the forefront of relevance. We believe that this adjustment will enhance the manuscript's clarity and pertinence for our readers.

- The manuscript's title suggests an exploration of the potential of LLMs in TKM, yet the article predominantly discusses LLM applications in the Korean licensure examination. I suggest either aligning the title with the article’s research question/methods/results/discussion and refining the manuscript content to better match the title.

**Answer:** In agreement with the editor's suggestion, we have revised the title as follows:

*“GPT-4 can pass the Korean National Licensing Examination for Korean Medicine Doctors”*

- While the introduction section touches on TKM, the article’s methods and results do not primarily focus on the differences in TKM questions. Strengthening the introductory paragraphs to create a clearer link between TKM and the examination context would enhance coherence.

**Answer:** We agree that it is needed to create a link between TKM and the examination.

**We added more introductory explanation about the examination in the method section (2.1. K-NLEKMD) as below:**

*“In the Republic of Korea, a TKM doctor must pass the K-NLEKMD under the administration of the National Health Personnel Licensing Examination Board (https://www.kuksiwon.or.kr/) and be licensed by the Minister of Health and Welfare to obtain legal status and practice. This examination evaluates the competency of a prospective TKM doctor, encompassing questions that assess competencies both as a TKM specialist and a general medical practitioner. It is developed by faculty members from medical colleges or universities, all of whom have at least 3 years of teaching experience in the field and have completed a questionnaire development workshop. Candidates must correctly answer at least 60% of the total questions to pass the national examination and at least 40% of questions for each subject (Table 1) [25]. The examination comprehensively evaluates a candidate’s ability to recall medical knowledge, interpret presented clinical data, and provide appropriate care for patients in each situation.”*

However, it's also true that the examination for TKM doctors doesn't encompass all aspects of TKM. We've addressed the limitations in gauging the model's performance as detailed below:

**Answer:** *“K-NLEKMD is designed to assess the skills of candidates on a diverse range of subjects. However, this examination, mainly targeting medical trainees, may not be appropriate for assessing competencies at the level of experienced clinicians. Also, the focus on closed-ended questions in this study offers a limited view of LLM's capability, without delving into the quality of its reasoning. Further study should consider applying expert evaluation to determine the suitability of LLMs for real-world clinical use. Although this study does not suggest that LLMs are ready for clinical use, it is significant in demonstrating the feasibility of applying LLMs in TKM with a certificated assessment of TKM knowledge and inference.”*

- Improvement is needed in the introduction and sections addressing sources of biases and limitations inherent to LLMs.

**Answer:** We have updated the title of the article by removing the term 'cultural-adapted medicine'. In the updated manuscript, we mainly focused on the performance of GPT-4 for application in TKM. The biases and limitations inherent to LLMs are addressed in the discussion section, where we delve into the discrepancies in performances.

We have improved the discussion about the inherent biases of LLM, including the added explanation as below:

*“Furthermore, biases in LLMs, which emerge from patterns in their training data, could potentially lead to health disparities and harm, especially in socioeconomic or racial contexts [40, 41], This is of particular concern for East Asians who might be underrepresented in datasets primarily from North America and Europe, and thus may be disproportionately affected by these biases. On the other hand, it's also worth noting that texts related to TKM or traditional Asian medicine predominantly originate from East Asian countries where Asians are the majority. This indicates the need for caution when using LLMs to make TKM inferences about non-Asian ethnicities. Such biases rooted in prior knowledge must be considered when applying transnationally developed LLMs to fields such as healthcare, including TKM.*

- If the questions are not publicly accessible, clarification is needed regarding the source of questions and answers. Additionally, information about any annulled questions or quality control measures would enhance transparency.

**Answer:** We added more information about the source of questions and answers as below:

*“The questions and answers are accessible upon request to the National Health Personnel Licensing Examination Board.”*

Also, we have described annulled answers with examples as below:

*“If the model refused to provide an answer because it was not authorized for medical diagnosis or prescription (ex. "I am an artificial intelligence model and do not have specialized medical knowledge or diagnostic abilities. You should consult a medical professional for a diagnosis.”), or because answering a test question would violate academic integrity (ex. “It is a violation of academic ethics to use a language model to solve test questions.”), the response was discarded and the question was resubmitted in a new session.”*

- Is there a comparative performance parameter between questions written in Chinese characters and those in Korean characters that supports the statement, “Considering that the language model may not be able to fully learn sentences containing TKM terms that are often written in Chinese characters but written only in Korean characters in the questionnaires”?

**Answer:** While we don't have direct evidence, we think this is a reasonable conclusion based on several facts. We can infer that documents produced in South Korea are likely to have a higher proportion of Chinese medical terms written in Chinese characters, as Koreans use either only Chinese characters or both Chinese characters and Hangul to describe Chinese medical terms. On the other hand, in China, only Chinese characters are used to record TCM terms, and the amount of TCM content produced in China is higher than in Korea. In particular, the Korean language, an ideographic script, has many homophones, while Chinese characters, an ideographic script, are free from homophone problems. Therefore, it is reasonable to assume that LLMs would be more familiar with Chinese content than Korean content.

Indeed, we found that Chinese-term annotation can contribute to the performance of accuracy as shown in Figure 1.

**We also revised sentences and paragraphs to reduce misleading.**

- If the manuscript aims to explore LLM capacity in TKM, an expansion of the description of TKM specialized knowledge questions and an analysis of their performance across different groups is suggested.

**Answer:** We have added more descriptions for the TKM specialized knowledge with examples as below:

*“Specifically, this examination evaluates candidates' proficiency as TKM specialists, alongside their general healthcare provision capabilities. To clarify, we anticipated that questions would require TKM specialized knowledge if they encompassed TKM etiological or staging concepts (like qi, yin, heat deficiency), treatment-related concepts (such as herbal medicine, acupuncture points), and specific TKM prescriptions (for instance, spleen-deficiency, so-gal, which parallels diabetes and its complications in Western medicine).”*

In addition, **new in this updated manuscript are Supplementary Figures 2-4,** which provide a deeper analysis of how association with TKM makes a difference in accuracy.

- Similarly, Table 2 could be divided to categorize TKM-specialized knowledge questions as yes/no and include the number of questions related to competency/table/images. Additionally, an analysis across TKM/non-TKM specialized knowledge questions (Figure 3) would enhance clarity.

**Answer:** We accept your suggestion and **have revised the table and the figures as shown in Table 2 and Supplementary Figure 2-4.**

- Regarding repeated questions, was a consistent answer required across all five repetitions for it to be considered final? Clarification is needed, especially considering the potential impact of an incorrect answer compared to a correct one. e.g., if the answer was 4 times correctly answered and 1 time wrong, was the answer considered wrong?

**Answer: We have revised as below:**

*“**When assessing the model's average capabilities, we typically had it tackle the same problem five times independently. We then calculated and reported the mean score and its standard deviation over these repetitions. To evaluate the performance based on self-consistency, we used the most frequent answer out of the seven repetitions for each question as the model's main response. Then, we graded it based on that.”*

So, the answer to your question would be: **the score would be 80% correct for the usual case (mean score), and considered right in the case of self-consistency.**

- I recommend including the number and details of questions encompassing TKM-specialized knowledge in the text, not solely within the table.

**Answer: We included the detailed numbers for the questions with more examples in the main text as below:**

*“For the 12 subjects: internal medicine (1) (focused on general internal diseases), internal medicine (2) (covering Shanghanlun and Sasang medicine, foundational theories in traditional Korean medicine), acupuncture medicine, public health & medicine-related law, dermatology & surgery, neuropsychiatry, ophthalmology & otorhinolaryngology, gynecology, pediatrics, preventive medicine, physiology (specialized in traditional Korean medicine), and herbology; there are 80, 32, 48, 20, 16, 16, 16, 32, 24, 24, 16, and 16 questions included respectively, as shown in Table 1. Furthermore, each question was labeled based on several criteria: the necessity of TKM-specialized knowledge for an answer (204 questions), the presence of a table (13 questions) or figure (46 questions), the subject from which the question originated, and the competency type it aimed to measure (114 questions for recall, 99 for diagnosis, and 127 for intervention). TKM-specialized knowledge is defined as knowledge that is intrinsic to TKM, distinguishing it from what other medical practitioners might know. Specifically, this examination evaluates candidates' proficiency as TKM specialists, alongside their general healthcare provision capabilities. To clarify, we anticipated that questions would require TKM specialized knowledge if they encompassed TKM etiological or staging concepts (like qi, yin, heat deficiency), treatment-related concepts (such as herbal medicine, acupuncture points), and specific TKM prescriptions (for instance, spleen-deficiency, so-gal, which parallels diabetes and its complications in Western medicine).”*

- The discussion about the lower performance of LLMs in TKM questions and the reasons discussed in the discussion section, such as limited depth in algorithm training data and insufficient scientific articles on TKM, should be expanded upon. Do the authors believe that applying LLM-based models as clinical assistants, for medical education, or TKM research would benefit or harm the Korean population?

**Answer:** To conclude, although GPT-4 passed the examination, we are concerned that the relatively low performance of LLM on TKM revealed in this study may be a barrier to using LLM directly for work on TKM. Therefore, in the revised manuscript, we have added a more specific discussion of this weakness.

**In the discussion section, we have revised and extended the reason for the low performance for TKM as below, focusing on the inherent cultural bias in the LLMs as below:**

*“In our study, we observed low accuracy when applying the model to public health and medicine-related law in Korea. Similarly, in the Japanese Medical Licensing Examinations, it was noted that LLMs strongly recommended against certain medical practices in Japan, including euthanasia [39]. Both cases suggest that the model may have difficulties in adapting to localized healthcare and medical policies. This underscores the importance of fine-tuning the model using datasets that reflect culture-specific medicine. Furthermore, biases in LLMs, which emerge from patterns in their training data, could potentially lead to health disparities and harm, especially in socioeconomic or racial contexts [40, 41], This is of particular concern for East Asians who might be underrepresented in datasets primarily from North America and Europe, and thus may be disproportionately affected by these biases. On the other hand, it's also worth noting that texts related to TKM or traditional Asian medicine predominantly originate from East Asian countries where Asians are the majority. This indicates the need for caution when using LLMs to make TKM inferences about non-Asian ethnicities. Such biases rooted in prior knowledge must be considered when applying transnationally developed LLMs to fields such as healthcare, including TKM.”*

Specifically, we have revised the discussions **for both the potential and concerns** in applying LLM-based models as clinical assistants, for medical education, or TKM research.

*“LLMs offer potential in clinical support, medical education, and TKM research. In clinical contexts, doctors should be able to discuss patients' symptoms with LLMs and receive guidance on medical decisions [42]. In the realm of medical education, LLMs can make resources more accessible, enhance resource efficiency, and offer simulation-based learning experiences [43]. For TKM research, LLMs can aid in understanding decision processes and address data collection challenges in clinical informatics. However, there are several concerns. Further research is essential for TKM-specific insights and ensuring real-world effectiveness. There's also a pressing need to mitigate the risk of LLMs providing outdated or biased answers. In education, the potential for conveying biases or incomplete medical information remains a concern. When used for TKM research, their inherent biases might misrepresent or overly simplify traditional practices and concepts.*

*.”*

In the conclusion of this paper, **we have emphasized the need for close collaboration between various professions** related to TKM and AI to compensate for the weak LLM in Korea-localized and TKM-specific areas.

*"However, it was observed that language-dependent performance degradation and limitations in Korea-localized and TKM-specific areas, underscoring the need for careful consideration in the safe application of LLM to TKM and other culturally-adapted medicines. For the safe and effective use of LLM in TKM, collaboration among healthcare providers, medical engineers, and policymakers is essential.”*

- It is common for medical exams to present straightforward and typical clinical scenarios that can potentially inflate LLM performance in healthcare. Does this phenomenon occur in the Korean licensure examination? Moreover, is LLM a reliable source of TKM information for Korean patients?

**Answer:** **We described the limitation that you mentioned in the discussion section as below:**

*“K-NLEKMD is designed to assess the skills of candidates on a diverse range of subjects. However, this examination, mainly targeting medical trainees, might not reflect the depth of understanding of experienced practitioners.”*

- I suggest adding to the discussion section about the risks associated with deploying LLM-reliant systems for Korean healthcare/TKM. Considering that LLMs are trained on less than 1% of Korean data, they pose a potential risk to the Korean population

**Answer:** **We have added more discussion as below:**

*“Furthermore, biases in LLMs, which emerge from patterns in their training data, could potentially lead to health disparities and harm, especially in socioeconomic or racial contexts [42, 43], This is of particular concern for East Asians who might be underrepresented in datasets primarily from North America and Europe, and thus may be disproportionately affected by these biases. However, it's also worth noting that texts related to TKM or traditional Asian medicine, which have a shared background, predominantly originate from East Asian countries where Asians are the majority. This indicates the need for caution when using LLMs to make TKM inferences about non-Asian ethnicities. Such biases rooted in prior knowledge must be considered when applying transnationally developed LLMs to fields such as healthcare, including TKM.”*

**Dear Reviewer 1,**

Throughout the paper, the authors directly embedded Korean questions into GPT 3.5 and GPT4.0 to solve the test questions.

As a result, the hypotheses of this study becomes a mixture of two topics.

First, how accurately do GPT 3.5 and GPT4.0 translate TKM-related questions in Korean or Korean+Chinese?

Second, how well do GPT 3.5 and GPT4.0 understand and solve TKM-related questions (assuming accurate translation)?

The two issues must be distinguished, otherwise, the purpose of this study will be obscured.

In the Discussion, the authors extrapolate the problem of language as the reason why LLMs scored lower on the K-NLEKMD's test.

This issue could be clarified by having GPT 3.5 and GPT4.0 translate the K- NLEKMD and then having TKM experts evaluate the quality of the translation.

If 5 to 10 representative questions per subject and per feature were selected for translation and evaluation, the authors would be able to provide accurate evidence rather than vague guesses.

Furthermore, if the same study were conducted with a sufficiently well-translated test paper in English, it would be possible to evaluate how well GPT 3.5 and GPT 4.0 are able to address TKM knowledge without prior training.

This is an example of how the two hypotheses that are currently conflated can be interpreted separately. Please refer to the above methods and present your hypotheses, methods, and results more clearly.

If you have difficulty translating the full text of the K-NLEKMD into English, I recommend that you make it clear in the first question; how much the translation problem may have affected the accuracy of the question.

**Answer:** We acknowledge your reservations. Broadly speaking, language models like GPT don't handle multiple languages by converting them to English; instead, they generate predictions based on the original language structure. That said, we concur that performance might vary depending on the language in which the input is presented. For our study, we inputted Korean directly into GPT without translating it. This might result in a different performance outcome compared to when the input is first translated into English. This is attributed to the fact that terms denoting similar concepts might be embedded differently based on the language. Given that the refinement of these LLM embeddings can differ across languages, it's plausible that translating the same term into English might yield superior performance.

Your observation ties closely with prompt engineering—a subject of curiosity for several other reviewers too. This nudged us to delve deeper into understanding how prompt engineering influences the model's output. **To that end, we explored the variations in performance across different prompt types, encompassing numerous factors we suspected could sway results, including the language of the input (whether Korean or English).**

**The findings of these experiments are predominantly showcased mainly in Figure 1 and Supplementary Table 1, and further detailed in the section '3.1. Overall performance on the K-NLEKMD'**.

For the main body of the paper, please consider the comments below for revision.

In section 3.1, the meaning of 'questions (native) without the labeling' is unclear, and it is unclear what 'former case' means. Please describe them more clearly.

**Answer:** We agree that our description was ambiguous. What you mentioned is about the Chinese-term annotation, and **the description was fully changed in the revised manuscript as below:**

*“We observed that the model's accuracy consistently improved as we introduced various techniques. Without applying any prompt techniques, the model achieved an accuracy of 51.82%. Meanwhile, annotating Chinese terms, translating the instruction and question into English, providing exam-optimized instructions, and utilizing self-consistency improved accuracy by 57.59%, 60.47%, 63.65%, and 66.18%, respectively (Figure 1). This indicates that each of these modifications plays a role in enhancing the model's accuracy.”*

In section 3.4, it is not clear how you distinguish between 'TKM specialized knowledge'. Please add what process you used to distinguish whether 'TKM specialized knowledge' was included in the question or not.

**Answer:** **We added more description in the 2.4. Classification for questions as below:**

*“TKM-specialized knowledge is defined as knowledge that is intrinsic to TKM, distinguishing it from what other medical practitioners might know. Specifically, this examination evaluates candidates' proficiency as TKM specialists, alongside their general healthcare provision capabilities. To clarify, we anticipated that questions would require TKM specialized knowledge if they encompassed TKM etiological or staging concepts (like qi, yin, heat deficiency), treatment-related concepts (such as herbal medicine, acupuncture points), and specific TKM prescriptions (for instance, spleen-deficiency, so-gal, which parallels diabetes and its complications in Western medicine).”*

It is also recommended to analyze the questions at least in three categories: 1) questions that ask for modern medical knowledge (for which sufficient performance is already known), 2) questions that ask for traditional Chinese medicine knowledge (for which this study actually wants to know), and 3) questions that mix traditional Chinese medicine and modern medical knowledge.

**Answer:** We appreciate your feedback. However, the number of questions that fall into the second of your cases is relatively small, and there is a large variation in the proportion of questions across subjects. Therefore, the average score for that category is not expected to be informative. Therefore, we have decided to keep the calculation as it is now, with only two cases: questions that are related to TKM and questions that are not.

**Dear Reviewer 2,**

Methodology:

The methodology section describes the dataset used for evaluation, the specific subjects and questions included in the test, the LLMs utilized (GPT-3.5 and GPT-4), and the evaluation process.

In section 2.1. K-NLEKMD: Since the prompts used for model training are not publicly available, authors must be more detailed about what the dataset is made up of. Details should be added such as how many questions there are for each existing competence or for each subject or if the different subdivisions have already been defined by the researchers or are already part of the exam.

**Answer:** Thank you for your insightful comments. The details you referred to, including the number of questions, are presented in Tables 1 and 2.

For the readers' convenience, **we have also provided the specifics in the 2.4. Classification for questions is as follows:**
*“For the 12 subjects: internal medicine (1) (focused on general internal diseases), internal medicine (2) (covering Shanghanlun and Sasang medicine, foundational theories in traditional Korean medicine), acupuncture medicine, public health & medicine-related law, dermatology & surgery, neuropsychiatry, ophthalmology & otorhinolaryngology, gynecology, pediatrics, preventive medicine, physiology (specialized in traditional Korean medicine), and herbology; there are 80, 32, 48, 20, 16, 16, 16, 32, 24, 24, 16, and 16 questions included respectively, as shown in Table 1. Furthermore, each question was labeled based on several criteria: the necessity of TKM-specialized knowledge for an answer (204 questions), the presence of a table (13 questions) or figure (46 questions), the subject from which the question originated, and the competency type it aimed to measure (114 questions for recall, 99 for diagnosis, and 127 for intervention).”*

Results:

The results section presents the performance of GPT-3.5 and GPT-4 on the medical test. It reports accuracy scores for both models, highlighting that GPT-4 achieved near-passing performance. The analysis of performance across subjects, competency types, and the impact of tables and images on accuracy provides valuable insights. However the paper presents the accuracy results of GPT-3.5 and GPT-4 without comparing them to any baseline, values such as the average performance of test applicants or existing AI approaches in the field of TKM (if they exist).

**Answer:** We have found no prior studies or models specifically addressing the problem of national board examinations for oriental medicine doctors (including TKM), making direct comparisons challenging. Other language models requiring fine-tuning are not deemed suitable for comparison in this context. Furthermore, biomedical models like BioGPT may not be appropriate comparators, as they lack familiarity with both TKM and the Korean language. However, we have incorporated publicly available data regarding the average scores of human applicants in the manuscript and Figure 1 for reference.

**In the 3.1. Overall performance on the K-NLEKMD, we have described as below:**

*“**In the examination for the same questions for candidates for the TKM doctors, the average total accuracy was 76.7%, suggesting that the performance of GPT-4 on TKM is not yet at the level of human experts.”*

We note that **the updated prompt has improved the performance of the model as much as the model can pass the examination.**

Discussion:

The discussion interprets the results and offers potential explanations for variations in accuracy. It acknowledges the limitations of the models, particularly in handling TKM-specific knowledge, and points to the need for culturally-adapted training data to improve performance.

Cultural Bias: The paper mentions that GPT-4 showed limitations that were believed to be caused by culturally-biased learning. However, the specific examples or instances of cultural bias in the model's responses are not elaborated upon. Providing concrete examples of such biases would strengthen the paper's argument and highlight the need for culturally-adapted training data.

**Answer:** Thank you for your advice.

**We have described more examples for cultural biases of large language models below:**

*“(…), implying the culturally biased output toward Western culture from LLMs observed in several studies [36, 37] is due to the biased train dataset.”*

*“Furthermore, biases in LLMs, which emerge from patterns in their training data, could potentially lead to health disparities and harm, especially in socioeconomic or racial contexts [40, 41]. This is of particular concern for East Asians who might be underrepresented in datasets primarily from North America and Europe, and thus may be disproportionately affected by these biases.”*

The paper uses questions from the 2022 K-NLEKMD for the evaluation of GPT-3.5 and GPT-4. However, there is no discussion about the representativeness of this dataset in terms of the diversity and complexity of Traditional Korean Medicine. The authors should justify why this particular dataset was chosen and discuss potential biases or limitations associated with it.

**Answer:** We acknowledge your concern regarding the representativeness of the examination for the field of medicine. In essence, the K-NLEKMD is managed by a government organization, and candidates must pass this examination to attain legal practicing status. This assessment is crafted to evaluate a broad spectrum of abilities and is administered by medical education experts and esteemed professors from each subject within the College of Korean Medicine. Additionally, it's worth noting that, to the best of our knowledge, no reliable Q&A dataset for TKM exists apart from the K-NLEKMD.

**We have updated the introduction in the 2.1. K-NLEKMD accordingly:**

*“In the Republic of Korea, a TKM doctor must pass the K-NLEKMD under the administration of the National Health Personnel Licensing Examination Board (https://www.kuksiwon.or.kr/) and be licensed by the Minister of Health and Welfare to obtain legal status and practice. This examination evaluates the competency of a prospective TKM doctor, encompassing questions that assess competencies both as a TKM specialist and a general medical practitioner. It is developed by faculty members from medical colleges or universities, all of whom have at least 3 years of teaching experience in the field and have completed a questionnaire development workshop. Candidates must correctly answer at least 60% of the total questions to pass the national examination and at least 40% of questions for each subject (Table 1) [25]. The examination comprehensively evaluates a candidate’s ability to recall medical knowledge, interpret presented clinical data, and provide appropriate care for patients in each situation.”*

We, however, also acknowledged that K-NLEKMD may not fully encompass the actual TKMs realized in the clinic.

**We described the limitations of our benchmark method in the discussion section as below:**

*“K-NLEKMD is designed to assess the skills of candidates on a diverse range of subjects. However, this examination, mainly targeting medical trainees, may not be appropriate for assessing competencies at the level of experienced clinicians. Also, the focus on closed-ended questions in this study offers a limited view of LLM's capability, without delving into the quality of its reasoning. Further study should consider applying expert evaluation to determine the suitability of LLMs for real-world clinical use. Although this study does not suggest that LLMs are ready for clinical use, it is significant in demonstrating the feasibility of applying LLMs in TKM with a certificated assessment of TKM knowledge and inference.”*

Conclusion:

The performance of the model do not seem to be entirely encouraging in some of the tests, demonstrating how in some cases the use of models such as GPT-3.5 or GPT-4 can also be harmful for the health systems of underrepresented groups. Both in the discussion and in the conclusions, the risks of these biases must be mentioned.

The conclusion should include a more comprehensive discussion of future directions for research. This could involve addressing the identified limitations, exploring ways to mitigate cultural biases, and proposing potential collaborations with experts in Traditional Korean Medicine for fine-tuning and enhancing the model's performance.

**Answer:** **We discussed the potential harms in the discussion section as below:**

*“In our study, we observed low accuracy when applying the model to public health and medicine-related law in Korea. Similarly, in the Japanese Medical Licensing Examinations, it was noted that LLMs strongly recommended against certain medical practices in Japan, including euthanasia [39]. Both cases suggest that the model may have difficulties in adapting to localized healthcare and medical policies. This underscores the importance of fine-tuning the model using datasets that reflect culture-specific medicine. Furthermore, biases in LLMs, which emerge from patterns in their training data, could potentially lead to health disparities and harm, especially in socioeconomic or racial contexts [40, 41]. This is of particular concern for East Asians who might be underrepresented in datasets primarily from North America and Europe and thus may be disproportionately affected by these biases. On the other hand, it's also worth noting that texts related to TKM or traditional Asian medicine predominantly originate from East Asian countries where Asians are the majority. This indicates the need for caution when using LLMs to make TKM inferences about non-Asian ethnicities. Such biases rooted in prior knowledge must be considered when applying transnationally developed LLMs to fields such as healthcare, including TKM.”*

Also, **we re-write the conclusion as below:**

*“In conclusion, this study offers early findings on the application of LLM to TKM. We found that GPT-4 achieved performance that is enough to pass the K-NLEKMD without advanced training for TKM, suggesting the potential of the application of LLM to TKM. However, it was observed that language-dependent performance degradation and limitations in Korea-localized and TKM-specific areas, underscoring the need for careful consideration in the safe application of LLM to TKM and other culturally-adapted medicines. For the safe and effective use of LLM in TKM, collaboration among healthcare providers, medical engineers, and policymakers is essential.”*

**Dear Reviewer 3,**

Strengths:

1) Paper is well-written and easy to read

2) Authors performs analyses from different viewpoints (e.g., sensitivity, consistency, subgroup performance)

3) Authors consider how answers are scored: e.g., in borderline cases where option may not be correctly mentioned, questions contain text/images

4) Authors consider if the questions are present in the training data

**Answer:** Thank you for your comments.

Weaknesses:

1) The protocol for marking answers that do not answer with the option number correctly is confusing: how is it judged to be "similar enough" to the correct answer?

**Answer:** We think that the writing in the manuscript was a little confusing. Each question has five choices and only one of them is the correct answer. If the answer of the language model is not the same as the correct answer, it is counted as the wrong answer. The model predicts the answer with five repetitions independently and we present the mean accuracy of the prediction.

**We have revised the manuscript in the 2.5. Encoding of answers as below:**

*“Each question has five choices and only one of them is the correct answer. If the response of the language model is not the same as the correct answer, it is counted as the wrong response.”*

*“When assessing the model's average capabilities, we typically had it tackle the same problem five times independently. We then calculated and reported the mean score and its standard deviation over these repetitions. To evaluate the performance based on self-consistency, we used the most frequent answer out of the seven repetitions for each question as the model's main response. Then, we graded it based on that.”*

2) It is not clear how images are passed to the model -- perhaps an illustrative image/overall flowchart would help?

**Answer:** Images are not input to the model. The GPT-4 model used in the experiment cannot handle images as input. Therefore, only text without images was input. Nevertheless, the percentage of correct responses to these questions was not significantly different from the questions without images, suggesting that text alone was sufficient to solve the problem.

**We have revised the writing for more clearer explanation in 2.2 Language models and inputs as below:**

*“For questions that included images, we excluded the images from the input and used only the accompanying text, since our version of the model couldn't process images at the time of the experiment .”*

3) The rationale for the specific prompt chosen (i.e., "..for research purposes...") is not clear. Have the authors experimented with additional prompts?

**Answer:** In the case of the sentence in the prompt that you mentioned ("These results are for research purposes only ") is added to reduce the probability for the model to refuse to answer questions that would be related to medical purposes and academic cheating.

We agree with your advice and we think that this is very important in this study. **We have processed additional experiments and presented the results in the manuscript.** **The more details of the specific prompts are described in the Figure 1, Supplementary Table 1, and the ‘3.1. Overall performance on the K-NLEKMD’ in the main manuscript.**

We note that the updated prompt has improved the performance of the model as much as the model can pass the examination.
